# Antônio Austregésilo Rodrigues de Lima (1876–1960) on his 150th anniversary: the polymath of Brazilian neurology

**DOI:** 10.1055/s-0046-1827167

**Published:** 2026-08-13

**Authors:** Marleide da Mota Gomes, Marcos Raimundo Gomes de Freitas

**Affiliations:** 1 Universidade Federal do Rio de Janeiro, Faculdade de Medicina, Departamento de Medicina Interna, Rio de Janeiro RJ, Brazil.; 2 Universidade Federal do Rio de Janeiro, Instituto de Psiquiatria, Programa de Pós-Graduação em Psiquiatria e Doenças Mentais, Rio de Janeiro RJ, Brazil.; 3 Universidade Federal Fluminense, Faculdade de Medicina, Departamento de Medicina Interna, Niterói RJ, Brazil.

**Keywords:** Brazil, History, 20th Century, Neurology, Biography, Ethics, Medical

## Abstract

The present study reexamines the enduring impact of Antônio Austregésilo Rodrigues de Lima (1876–1960), a pioneer who shaped Brazil's medical landscape. Austregésilo did not just introduce European medical traditions—particularly those of Jean-Martin Charcot and Emil Kraepelin—to Brazil; he reimagined them to address the country's unique struggles with infectious diseases, weak institutions, and rapid social change. His work spanned clinical practice, political advocacy, literature, and education, revealing a career defined by innovation and a relentless drive to professionalize medicine. Yet, Austregésilo's legacy is not without its contradictions. While he helped legitimize neurology in Brazil, his contributions also reflect the ethical, social, and gender biases of his time. This paper argues that his story is ultimately one of creative adaptation—a testament to how global medical knowledge is reshaped within the realities of a postcolonial society.

## INTRODUCTION

On the sesquicentennial of Antônio Austregésilo Rodrigues de Lima's birth, this study critically examines his pioneering yet complex legacy in Brazilian medicine and society. Too often, Austregésilo's contributions have been reduced to idealized narratives—what scholars call hagiography (a reverential, uncritical portrayal of historical figures, akin to saintly biographies). Yet, his multidisciplinary work remains underexplored, especially in how it shaped—and was shaped by—the challenges of early 20 ^th^ century Brazil.

Austregésilo institutionalized neurology in Brazil in the first half of the 20th century—a task that, while echoing the challenges faced by pioneers in European medical centers, was shaped by the distinct realities of a young republic burdened by tropical diseases, scarce resources, and a fledgling medical infrastructure. This study aims to understand how he adapted European models to Brazil's unique realities. As well as what ethical and social tensions emerged from his interdisciplinary approach. For a concise overview of his life and career, see Table 1 . 1 2 3 4 5 6 7

**Table 1:** Antônio Austregésilo Rodrigues de Lima (1876–1960) – Biographical and Professional Summary 1 2 3 4 5 6 7

Category	Details	Notes & Elaborations
**Birth & Death**	Born April 21, 1876, in Recife, PE. Died December 23, 1960, in Rio de Janeiro, RJ.	Overcame humble origins, mulatto background, a stutter, and childhood tics through relentless dedication, described as he “studied like a slave.”
**Family**	Son of lawyer José Austregésilo Rodrigues de Lima and Maria Adelaide Feitosa Lima. Over two marriages (20 and 7 years), he fathered six children: four with his first wife and two with his second. Among them was his son, Antônio de Moraes Austregésilo Filho (1904–1954), a pioneering Brazilian neurologist, professor, and member of the Academia Nacional de Medicina.	Educated at Colégio das Artes in Recife, where he engaged early with the “Escola de Recife” literary movement.
**Education**	MD, Faculty of Medicine of Rio de Janeiro (1899). Thesis: “Estudo Clínico do Delírio”.	Influenced by Professor Francisco de Castro. Served as a medical aide during the 1896 cholera epidemic in Vale do Paraíba.
**Key Academic & Clinical Positions**	• Professor of Neurology, Faculty of Medicine of Rio de Janeiro (UFRJ, 1912–1944), later Professor Emeritus. • Chief, Pinel Section, Hospício Nacional. • Head, Neurology Clinic, Santa Casa da Misericórdia Hospital. • Co-founder, Sanatório Botafogo. • Director, “Arquivos Brasileiros de Neurologia e Psiquiatria” and Instituto de Neuropatologia.	Considered the founder of Brazilian Neurology. He was appointed substitute professor in 1909, and professor in 1912.
**Major Scientific Works**	• **1912:** Codescription of the 'Austregésilo-Esposel sign' (a substitute for the Babinski sign). • **1916:** Introduction of the concept of “Catafrenia” (acquired, reversible mental weakening). • *Clínica Neurológica* (3 vols., 1917, 1934, 1945): Foundational textbooks. • **1919:** *Psiconeuroses e Sexualidade* (advocated psychotherapy as reeducation of the will). • **1927:** *Troubles nerveux et mentaux dans les maladies infectieuses* (based on his Sorbonne lectures). • **1936:** *L'analyze mentale dans les psychoneuroses* (published by Masson, Paris). • **1940:** *Manuel de psychothérapie pratique* (Masson). • **1945:** Pioneered the atropinic treatment for Parkinsonian syndrome in Brazil. • Numerous papers on neuromyelitis, neuroviroses, frontal lobe syndromes, etc.	Work bridged neurology, psychiatry, and internal medicine. He was an early researcher in neurotropic infections, deficiencies, and movement disorders. His psychotherapeutic approach integrated moral, philosophical, and biological perspectives.
**Disciples & Legacy in Neurosurgery**	Alfredo Alberto Pereira Monteiro, José Ribeiro Portugal.	After visiting Harvey Cushing and Charles Frazier in the USA (1928), he formally tasked these surgeons with establishing what became the Brazilian School of Neurosurgery.
**Honors & Memberships**	• **Honors:** Legion of Honor (France); Order of Santiago da Torre e Espada (Portugal); Order of the Crown (Romania). • **Memberships:** President, Academia Nacional de Medicina (1934–1937, 1945–1947, 1949–1951). Member of the Brazilian Academy of Letters (President in 1939). Member of the Lisbon Academy of Sciences. Honorary member of numerous national and international medical societies.	Recognized globally as a scientific and cultural leader. Represented Brazil at multiple International Congresses of Neurology and Psychiatry.
**Literary & Humanistic Works**	• **1898:** *Manchas* (poetic prose). • **1921:** *Educação da Alma* (Education of the Soul). • **1924:** *Perfil da Mulher Brasileira* (Profile of the Brazilian Woman). • **1943:** *Perfis de Loucos* (Profiles of the Mad - psychological studies of psychosis). • **1950:** *Vidas Desgraçadas* (Wretched Lives - his only novel). • Many essays on psychology, spirituality, and character.	His literary style leaned toward Symbolism. He consistently blended medical insight with philosophical, social, and literary reflection.
**Political Role**	Federal Deputy for Pernambuco (1922–1930).	Advocated for public health and educational reforms during his legislative tenure.
**Overall Legacy**	Founded the first school of neurology in Brazil and was the seminal catalyst for the birth of Brazilian neurosurgery. His influence persists through major institutions, a substantial literary corpus, and the generations of specialists he trained.	His work formed the foundation for the modern Institute of Neurology (UFRJ). The main auditorium (Salão Nobre) of the Brazilian National Academy of Medicine bears his name.

## AUSTREGÉSILO'S LEGACY: BRIDGING NEUROLOGY, HUMANISM, AND PSYCHOTHERAPY

Austregésilo's career was rooted in the neuropsychiatric traditions of Brazilian medicine, where the study of “Mental and Nervous Diseases” remained an integrated field. 8 9 Far from merely inheriting this legacy, he ushered in a transformative era for Brazilian neurology, reshaping its scope through innovation, interdisciplinary collaboration, and a vision aligned with the country's distinct needs.

Initially trained by Francisco de Castro and Miguel Couto, he gained a strong foundation in local clinical practice. 1 However, it was his partnership with Juliano Moreira at the National Hospice for the Insane that proved most influential, as Moreira's advocacy for compassion as a therapeutic approach and his resistance to bureaucratic constraints profoundly shaped Austregésilo's conviction that ethical leadership was essential to scientific advancement. 9 This commitment to rigor and precision also extended to his clinical work, as seen in his 1908 study “ *Histeria e síndrome histeroide* ”, where he distinguished true hysteria (pitiatism), rooted in autosuggestion and curable by persuasion, from hysteroid syndrome, which mimicked hysteria but often masked underlying organic or functional disorders (e.g., dementia praecox, tumors, or syphilis). 10 His approach aligned with Babinski's efforts to redefine this syndrome as one grounded in psychological mechanisms.

His work represented a dynamic synthesis of European scientific knowledge and Brazilian realities. Austregésilo adapted neurology to address local challenges, creating the Austregésilo-Esposel sign (1912), 11 providing one of the world's earliest descriptions of posttraumatic dystonia, 12 and introducing Parkinson's disease treatments to Brazil. 13 He also applied controversial frameworks like 'degeneracy', a mainstream European theory of his era advanced by Morel and Lombroso, to study tropical diseases, from influenza to Chagas disease. 14 15 His focus on syphilis—captured in the phrase “One must think syphilitically”—bridged European psychiatry with Brazil's infectious disease landscape.

Austregésilo developed a distinctive approach to psychotherapy in Brazil. 16 Though he recognized Freud's contributions and oversaw the country's first psychoanalytic thesis, his method centered on “mental reeducation,” emphasizing habit modification and cognitive realignment through rational persuasion and therapeutic suggestion. His approach drew more from Paul Dubois and Joseph Babinski than from Freudian theory, positioning him as a forward-thinking figure in Brazilian psychotherapy.

Dubois' rational persuasion technique—detailed by Corrêa 16 as a logical appeal to address nervous disorders—reflected Stoic principles, such as confronting irrational beliefs. While Dubois did not directly shape Cognitive Behavioral Therapy (CBT), his philosophical and methodological alignment with its core tenets paralleled Austregésilo's own innovative contributions.

Austregésilo's legacy endures at the intersection of neurology, tropical medicine, and psychotherapy, reflecting both his scientific brilliance and the ethical dilemmas his work raises. His story is a testament to how medical knowledge is localized, contested, and reinvented in response to a nation's unique challenges.

## THE INTERNATIONAL NEUROLOGIST

Austregésilo engaged with the foremost neurological circles in Europe, where he interacted with some of the most prominent neurologists of his time. 7 This experience was pivotal in shaping his professional development and enabled him to bring the most advanced knowledge in the field back to Brazil. He interacted with leading figures in neurology, including Joseph Babinski, Georges Guillain, Fedor Krause, Hermann Oppenheim, Charles Foix, Pierre Marie, and Gordon Holmes. At a meeting in Hamburg, he connected with the distinguished English neurologist Samuel Alexander Kinnier Wilson—a pivotal encounter where he presented his theories, seeking both validation and intellectual exchange. This underscores his commitment to integrating Brazilian neurology into the global scientific dialogue and his ambition to earn recognition within the international neurological community.

Austregésilo's engagement with international neurology was not passive reception but active intellectual exchange. His command of international respect was best evidenced by the December 1952 issue of The Journal of Nervous and Mental Disease. In an issue featuring contributions on Robert Wartenberg, the Neurological School of Vienna, and biographies of Gowers and Cushing, Austregésilo Filho's article on “Austregésilo and Brazilian Neurology” appeared alongside these historical figures—signaling that Brazilian neurology, and Austregésilo's foundational role in it, was regarded as part of the global neurological heritage. 17

Yet Austregésilo's internationalism was never mere mimicry. He brought to Europe a perspective shaped by Brazil's unique medical realities—tropical diseases, nutritional deficiencies, Chagas disease—enriching global neurological discourse with observations from the periphery. His career exemplified how scientific knowledge flowed not only from centers to peripheries but could be reimagined and returned, transformed by local experience.

## PHYSICIAN-STATESMAN, EDUCATOR, AND INSTITUTION-BUILDER

Austregésilo's influence extended to literature, politics, and education. 3 Elected to the Brazilian Academy of Letters in 1914, he merged medicine with Symbolist aesthetics in works like Educação da Alma (1921), Manchas (1898), Perfis de Loucos (1943), and Vidas Desgraçadas (1950). As the 3-time president of the National Academy of Medicine (1934–1937, 1945–1947, 1949–1951), 1 his reforms modernized Brazilian medicine but also perpetuated class and gender biases. In “Perfil da Mulher Brasileira” (1923), he argued that women's progress required adopting male intellectual standards, reflecting essentialist views. 18

**Figure 1:**
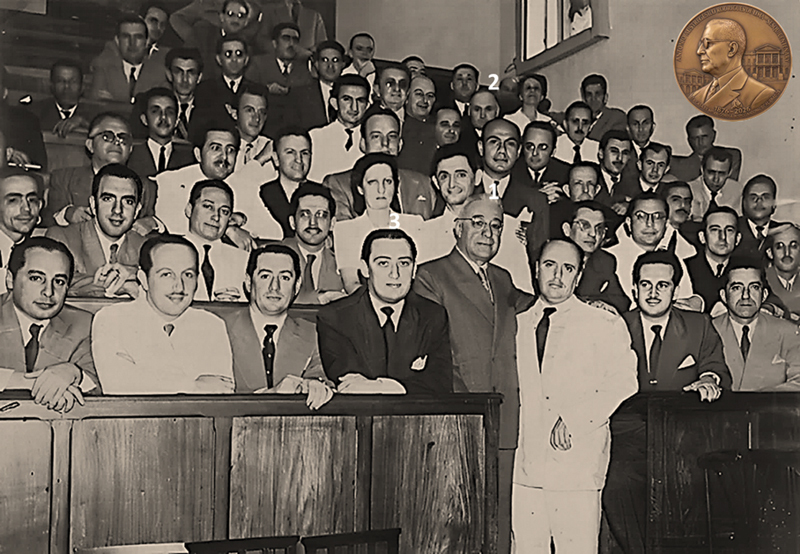
Source: Gomes and Costa. 20 Austregésilo's 1944 Retirement Lecture – A Landmark in Brazilian Neurology. This 1944 photograph captures Austregésilo delivering his retirement lecture at Santa Casa da Misericórdia in Rio de Janeiro, a historic moment marking the culmination of his 32-year mission (1912–1944) to establish neurology as a formal academic discipline in Brazil. The image encapsulates the institutional legacy and generational transition of the neurology chair: ( **1** ) Austregésilo stands at the center, flanked by ( **3** ) Bernardo Couto seated to his right and ( **2** ) Deolindo Couto behind him, underscoring the passing of leadership – and subtly reflecting the notable absence of women in academia at the time. Also present are influential figures such as Paulo Elejade and Antônio Rodrigues de Mello to Austregésilo's left, along with Abraham Akerman and José Ribeiro Portugal. The 2026 sesquicentennial medal inset pays tribute to Austregésilo's enduring contributions while framing them within the scientific and social paradigms of his era.

His 1944 retirement lecture ( Fig. 1 ) symbolized his multidisciplinary school, though it also critiqued the era's exclusionary practices. His disciples disseminated his methods nationally, supported by over 50 works, including *Clínica Neurológica* , cited internationally. 5 Pioneering studies on movement disorders 13 and infectious neurology 14 15 adapted Kraepelin's models to local psychoses, 14 but concepts like “degeneracy” and gendered perspectives revealed ethical limits.

Austregésilo's teaching style cultivated critical thinking in his students. He especially valued those with open, exploratory minds, free from dogma—students he affectionately called jejunos.

At Santa Casa de Misericórdia Hospital, his lessons in the 20 ^th^ ward and outpatient clinics became legendary. Combining precision with academic rigor, he built his pedagogy around patient-focused discussions. A hallmark of his method was the “diagnostic auction,” held every Wednesday in outpatient clinics. This exercise turned clinical reasoning into a dynamic, interactive, and often daunting, challenge. 7

His disciple, Antônio Rodrigues de Mello, later recounted Austregésilo's guidance: “Write many papers, but only 1 in 10 will truly matter.” 19 Outside the classroom, he was known for his love of dance. This portrait drew from Mello's personal recollections, shared directly with the authors of the current study.

Austregésilo died in Rio de Janeiro on December 23, 1960, with his disciple, Mello, present, underscoring his enduring impact through mentorship and institution-building.

## CONCLUSION

Austregésilo was a foundational yet deeply complex figure in Brazilian medicine. His career exemplifies how global medical knowledge was creatively adapted to the pressing realities of a postcolonial society. As the present study has shown, his work bridged neurology, psychiatry, and psychotherapy, while his institutional leadership and international engagement helped establish Brazilian neurology as a legitimate field within the global scientific community.

At the same time, Austregésilo's legacy cannot be disentangled from the ethical and social contradictions of his era. His contributions—whether in clinical innovation, medical education, or literary production—were shaped by the hierarchies of class, gender, and race that structured early 20th-century Brazil. His vision of progress often reinforced exclusionary norms, even as he pushed against institutional inertia.

Rather than offering a hagiographic portrait, this study positions Austregésilo as a figure whose achievements and limitations together illuminate the broader dynamics of medical modernization in Brazil. His story is one of innovation entangled with convention—a testament to how scientific authority is built, contested, and transformed at the margins of global medicine.
